# Revisiting cardiac regeneration: the role of remote heart regions after MI

**DOI:** 10.1093/procel/pwaf093

**Published:** 2025-11-05

**Authors:** Smin L Liu, Li Qian

**Affiliations:** Department of Pathology and Laboratory Medicine, McAllister Heart Institute, School of Medicine, University of North Carolina at Chapel Hill, Chapel Hill, NC 27599, United States; Department of Pathology and Laboratory Medicine, McAllister Heart Institute, School of Medicine, University of North Carolina at Chapel Hill, Chapel Hill, NC 27599, United States

Myocardial infarction (MI) remains a leading cause of morbidity and mortality worldwide, and surviving patients are at high risk of developing heart failure due to the heart’s limited regenerative capacity (Martin et al., 2025). Most research on heart injury after MI focuses on the part of the heart directly damaged. With the emergence of multi-omic and spatial transcriptomic technologies, it has become increasingly feasible to map regional cellular responses and signaling across the injured and remote myocardium, offering new insight into organ-level coordination during repair. Therapies under investigation include transplantation of pluripotent stem cell-derived cardiomyocytes, delivery of growth factors such as neuregulin-1 or IGF-1/HGF, introduction of microRNAs and transcription factors to stimulate cardiomyocyte proliferation, and modulation of cell cycle regulators to promote regeneration within the infarcted tissue (Bersell et al., 2009; Heallen et al., 2011; Liu et al., 2018; Mohamed et al., 2018). However, remote regions of the heart, such as the endocardium distant from the injury site, also respond to injury but remain comparatively understudied.

A recent discovery by Fan *et al.* provides evidence that remote regions of the heart contribute to repair and remodeling in ways that are not yet fully understood. It touches on a dimension rarely explored in mammalian heart research, providing an entry point for exploring organ-level regulatory mechanisms in mammalian systems. *Lyz2*, previously considered mainly as a myeloid immune marker and a gene whose promoter is widely used to target the myeloid lineage for genetic manipulation (Cross et al., 1988), is unexpectedly induced in endocardial endothelial cells (eECs) that are far from the injury site. This suggests that cardiac repair involves coordinated responses in the non-infarct area. Consequently, modulating this “remote response” may represent a novel therapeutic avenue to improve functional recovery after MI, which is especially relevant given the limited regenerative capacity of the adult human heart.

Using spatial transcriptomics and single-cell RNA sequencing, Fan *et al.* mapped gene expression in regenerative (P1) and non-regenerative (P7) hearts after injury. The team found high levels of Lyz2 not only at the infarct site but also in remote zones in the left ventricular endocardium (LVEZs). Notably, following myocardial injury, a cluster of cells marked by *Lyz2* emerged transiently. This population, which constituted ∼4% of all spots, was observed from 2 to 4 days post-injury before becoming undetectable by 5 to 7 days. This cell cluster was observed within both the apex injury region and the remote LVEZs. Its absence in the sham-operated controls confirms that its emergence is an injury-induced phenomenon.

Mechanistically, Lyz2 functions as a positive regulator of lysosomal degradation in endocardial cells. In human-induced pluripotent stem cell-derived endocardial-like cells, overexpression of *Lyz2* enhanced lysosomal activity and promoted ECM degradation, especially of heparan sulfate proteoglycans (HSPG). The induction of *Lyz2* by conditioned medium from injured cardiomyocytes further suggests a paracrine signaling mechanism in which secreted injury cues, possibly including cytokines or extracellular vesicle-borne factors, trigger this catabolic activity in the endocardium.

To confirm the detrimental role of Lyz2-mediated activity, the authors performed loss-of-function experiments. In a co-culture system, knocking down *Lyz2* in human endocardial-like cells preserved ECM (HSPG) integrity and protected adjacent cardiomyocytes from apoptosis, demonstrating that Lyz2-driven degradation of the ECM ultimately leads to cardiomyocyte death. This protective effect was recapitulated in vivo, where *Lyz2* knockout mice subjected to MI exhibited reduced levels of cardiomyocyte apoptosis, ECM degradation, and adverse remodeling. These structural improvements resulted in a significant functional benefit, with the knockout mice exhibiting a rapid and sustained recovery of cardiac function, including a significantly improved ejection fraction that persisted for at least 28 days.

To further validate these findings, the study employed pharmacological inhibitors of lysosomal degradation, CA-074 and Apilimod, in both *in vitro* and *in vivo* settings to observe responses after injury. Consistent with the genetic data, treating human endocardial-like cells with these inhibitors preserved HSPG and reduced apoptosis in co-cultured cardiomyocytes. This protective effect was confirmed *in vivo*, where administration of the inhibitors to mice following MI attenuated cardiomyocyte death, reduced fibrosis, and led to a significant improvement in heart function, including a higher ejection fraction.

This work emphasizes the importance of examining the relationship and synergistic potential of two core strategies in cardiac repair: cardiomyocyte protection strategies and cardiomyocyte regeneration strategies. Establishing a theoretical foundation that integrate “protection” with regeneration may enable the development of therapies that first stabilize the cardiac microenvironment and subsequently introduce reprogramming or pro-proliferation factors to regenerate the myocardium.

The discovery of a non-immune role for LYZ2 in the endocardium is particularly interesting when compared with other research on non-cardiomyocyte contribution to cardiac repair. For example, a recent study by Song *et al.* (2022) investigated the role of AMPKγ2, a protein highly expressed in the spleen and macrophages, in the context of post-MI outcomes. Using the *Lyz2*-Cre mouse model to delete *Ampkγ2* in the myeloid lineage, they found that its absence exacerbated cardiac dysfunction by promoting macrophage infiltration and inflammation (Song et al., 2022). The AMPKγ2 abstract keeps Lyz2 in its more traditional context, while Fan *et al.* redefined Lyz2 as acting in endocardial cells, where it drives ECM degradation. Together, these studies illustrate that both structural remodeling and immune inflammation need to be controlled to optimize post-MI recovery. In the long term, integrating remote zone protective strategies with regenerative reprogramming could rephase post-MI therapy, guiding combinatorial approaches that both preserve existing tissue and promote functional regeneration.

Despite the promising finding, the therapeutic translation of targeting *Lyz2* faces several significant challenges. Given that *Lyz2* is expressed in both myeloid and now eECs, systemic inhibition could have complex and potentially counterproductive effects on the post-injury immune response. Additionally, the lack of reliable biomarkers and methods to monitor LYZ2 activity *in vivo* complicates the assessment of its dynamic role and the development of therapeutic strategies. Future studies should investigate how to achieve spatiotemporal specificity in modulating lysosomal function and determine the optimal therapeutic window to maximize protective benefits while minimizing side effects.

Overall, this is a rigorous and forward-looking study. Beyond its specific mechanistic insights, its broader contribution lies in advancing the understanding of cardiac repair at the organ level. Defining how cross-regional regulatory mechanisms interface with cell fate-based strategies will be essential for advancing the next generation of therapeutic approaches in heart repair.

## Conflict of interest

Not applicable.

## Funding

Li Qian is supported by American Heart Association (20EIA35310348) and National Institute of Health/National Heart, Lung, and Blood Institute (R35HL155656).

## Ethics approval

Not applicable.

## Consent to participate

Not applicable.

## Consent for publication

Not applicable.

## Data Availability

Not applicable.
